# Analysis of the DXS Pan-Gene Family Reveals Evolutionary Conservation and Regulatory Divergence Underpinning Terpenoid Metabolism in Lauraceae

**DOI:** 10.3390/plants15182886

**Published:** 2026-09-21

**Authors:** Chao Fu, Siyang Tao, Shifang Wen, Xindong Wang

**Affiliations:** 1 Jiangxi Provincial Key Laboratory of Improved Variety Breeding and Efficient Utilization of Native Tree Species (NO. 2024SSY04091), Jiangxi Academy of Forestry, Nanchang 330032, China; plantfuchao@163.com (C.F.); taosy0822@163.com (S.T.); wenshifang@jxlky.cn (S.W.); 2 College of Forestry, Jiangxi Agricultural University, Nanchang 330045, China

**Keywords:** *DXS*, Lauraceae, pan-genome, whole-genome duplication, monoterpenoid biosynthesis

## Abstract

1-Deoxy-D-xylulose 5-phosphate synthase (DXS) catalyzes the rate-limiting step of the plastidial 2-C-methyl-D-erythritol 4-phosphate (MEP) pathway and governs carbon flux toward terpenoid biosynthesis. The Lauraceae constitute an economically important plant lineage characterized by specialized terpenoid metabolism and divergent essential-oil chemotypes; however, the evolutionary dynamics and functional divergence of the *DXS* gene family across this family remain poorly understood. Here, we performed a comprehensive pan-genomic survey of the *DXS* family across 14 Lauraceae genomes, integrating phylogenomics, synteny analysis, selection-pressure assessment, structural-variation profiling, spatiotemporal expression profiling, and heterologous functional validation. We identified 92 *DXS* genes assigned to six orthogroups. *DXS2* and *DXS4* were conserved ohnologs likely derived from the Lauraceae-specific whole-genome duplication, whereas *DXS5* and *DXS6* underwent recent lineage-specific duplications. All *DXS* orthogroups were under purifying selection (*Ka*/*Ks* < 1), with divergent selection intensities among subclades within Clade 2. Structural variations in *DXS* genes were predominantly located in introns and untranslated regions rather than coding regions. Spatiotemporal expression profiling in *Camphora officinarum* revealed distinct tissue-specific expression patterns: *CoDXS2* was specifically expressed in developing fruits, *CoDXS4* exhibited flower-bud-specific expression, and *CoDXS6* was highly expressed in developing leaves and fruits. Notably, *CoDXS6* transcript abundance showed a strong positive correlation with monoterpenoid accumulation (*r* = 0.945, *p* < 0.001), and functional assays confirmed its canonical catalytic activity. Furthermore, *CoDXS6* exhibited 6.32- to 9.18-fold higher leaf expression in terpenoid-dominant chemotypes compared with phenylpropanoid-dominant chemotypes. Collectively, retention following recent whole-genome duplication, recent genome-specific duplications, and regulatory variations jointly drove the functional divergence of Lauraceae *DXS* genes, highlighting *CoDXS6* as a key contributor to leaf monoterpenoid biosynthesis in *C. officinarum*.

## 1. Introduction

Terpenoids constitute the largest and most structurally diverse class of plant natural products. They participate in herbivore defense, pathogen resistance, pollinator attraction, and adaptation to diverse abiotic stresses [1,2]. Economically, terpenoids are indispensable as food flavorings, pharmaceuticals, and industrial feedstocks. To date, over 80,000 distinct terpenoid structures have been characterized, all derived from C_5_ isoprene building units. The universal precursors dimethylallyl diphosphate (DMAPP) and isopentenyl diphosphate (IPP) are synthesized via two spatially separated pathways in higher plants. The plastid-localized 2-C-methyl-D-erythritol 4-phosphate (MEP) pathway originates from pyruvate and glyceraldehyde-3-phosphate. It proceeds through seven enzymatic reactions and primarily supplies precursors for monoterpenoids and diterpenoids, exemplified by gibberellic acid, a well-known diterpenoid plant hormone [3,4]. In comparison, the cytosolic mevalonate (MVA) pathway consumes two acetyl-CoA molecules in six sequential steps to generate IPP, which is further isomerized into DMAPP by isopentenyl-diphosphate delta-isomerase (IDI). This route predominantly fuels sesquiterpene and triterpene production [3,5]. Abscisic acid (ABA) is a terpenoid-related hormone generated from tetraterpenoid carotenoid cleavage. Although strict subcellular compartmentalization separates these two pathways, partial IPP/DMAPP exchange can occur under certain physiological conditions [6]. Such metabolic crosstalk contributes to the metabolic plasticity underlying enormous terpenoid structural diversity.

The first committed step of the MEP pathway is catalyzed by 1-deoxy-D-xylulose 5-phosphate synthase (DXS). This enzyme condenses pyruvate and glyceraldehyde-3-phosphate to produce 1-deoxy-D-xylulose 5-phosphate (DXP), with thiamine diphosphate (TPP) serving as an indispensable cofactor [7]. DXS belongs to the transketolase superfamily. Its N-terminal region harbors a TPP-binding domain characterized by diagnostic GDG and LNDN motifs, together with invariant catalytic residues for cofactor coordination and substrate conversion [7,8]. DXS functions exclusively as a homodimer. Dimerization depends on conserved α-helical FEELG motifs from two monomers that assemble to form the active-site cavity; monomer dissociation leads to complete loss of enzymatic activity [9,10]. In land plants, DXS is typically encoded by small multigene families. For example, *Arabidopsis thaliana* and *Oryza sativa* each contain three *DXS* paralogs grouped into three phylogenetically distinct clades with divergent biological roles [11,12,13]. Clade 1 members exhibit constitutive transcription, maintaining basal isoprenoid flux for photosynthetic pigments and phytohormone biosynthesis. Clade 2 homologs are strongly induced by biotic and abiotic stresses and divert precursors toward defensive specialized terpenoids. Most Clade 3 proteins are catalytically inactive, while a small subset contributes precursor supply for gibberellin and abscisic acid biosynthesis [10,13,14]. Despite whole-genome duplication (WGD) events expanding *DXS* copy number across multiple plant lineages, this ancestral three-clade architecture is largely conserved among angiosperms [13].

DXS acts as the rate-limiting enzyme of the MEP pathway and therefore governs carbon allocation toward plastidial-derived terpenoid production [15]. Consistent with this gatekeeper role, heterologous overexpression of Clade 2 *DXS* paralogs elevates monoterpenoid and diterpenoid yields in multiple plant species [16,17]. At the transcriptional level, *DXS* genes respond to both endogenous cues and external environmental signals, including light, circadian rhythms, phytohormones, and various stress stimuli [16]. For instance, UV radiation triggers *DXS* up-regulation in *Bixa orellana* and *Cuminum cyminum*, enhancing terpenoid accumulation to mitigate UV-induced oxidative damage [18,19]. High temperature, salinity, and elevated sugar availability similarly increase *DXS* expression and terpenoid production [20,21,22]. Biotic interactions also remodel *DXS*-dependent metabolism. Co-cultivation with *Streptomyces pactum* markedly activates *DXS* transcription and promotes tanshinone accumulation in *Salvia miltiorrhiza* hairy roots [23]. Methyl jasmonate treatment up-regulates *DXS* to support geraniol biosynthesis in *Catharanthus roseus*; this hormonal cascade also facilitates tanshinone production in *Codonopsis* hairy roots [24,25]. Beyond environmental and hormonal inputs, several transcription factor families, such as ERF, MYB, HSF, and WRKY, directly target *DXS* promoters, constructing a multilayered regulatory network that fine-tunes MEP pathway flux [26,27,28,29].

Plants of the Lauraceae family are well-known for producing abundant essential oils enriched in monoterpenoids, sesquiterpenoids, and phenylpropene derivatives. Over the past decade, whole-genome assemblies have become available for roughly twenty Lauraceae species, including *Camphora officinarum* [30,31,32], *Camphora longepaniculata* [33], *Camphora kanahirae* [34], *Cinnamomum chago* [35], Cinnamomum burmanni [36], Phoebe bournei [37], Litsea cubeba [38], Lindera aggregata [39], Lindera glauca [40], Lindera megaphylla [41], Persea americana [42], and others. Notably, multiple high-quality genome assemblies have been released for *C. officinarum* representing different essential-oil chemotypes [30,31,32]. Most existing genomic investigations have focused on downstream terpene synthase (TPS) gene families. These studies document prominent TPS family expansion driven by tandem duplication (TD) and segmental duplication (SD) [31,32,33,34,36,37,38,39]. Paralogous *TPS* copies further undergo neofunctionalization and subfunctionalization, modulated by transcriptional regulation, post-translational modification, and subcellular compartmentalization, which collectively underpin chemotype diversification [31,36,38,43].

In sharp contrast, the *DXS* gene family remains understudied within Lauraceae. Detailed characterizations are only reported for two species. In *L. cubeba*, seven *DXS* members include Clade 2 isoforms (*LcDXS3-LcDXS5*) that are proposed to modulate monoterpenoid accumulation in fruit pericarps [28,38]. In *C. burmannii*, the Clade 1 isoform *CbDXS1* is critical for leaf terpenoid biosynthesis [44]. Nevertheless, these previous works are restricted to individual species and lack a pan-genomic perspective. The expansion landscape of the *DXS* family across Lauraceae, the functional partitioning of the three phylogenetic clades, the patterns of sequence variation and synteny conservation, as well as the spatiotemporal and chemotype-specific expression dynamics, remain almost entirely uncharacterized.

Here, we present a comprehensive pan-genomic analysis of the *DXS* family in Lauraceae. We systematically identify *DXS* members, define orthogroups, profile structural variations (SVs), and perform synteny reconstruction to establish a robust phylogenetic framework and clarify the evolutionary origins of *DXS* isoform diversity. We further characterize transcriptional divergence of three *DXS* clades across different tissues and *C. officinarum* chemotypes. By integrating transcript-metabolite correlation analysis and multiple functional-validation assays, we pinpoint the key isoform governing leaf monoterpenoid biosynthesis. Our findings provide promising candidate genes and theoretical foundations for molecular breeding of terpenoid-rich Lauraceae germplasm, as well as for synthetic-biology chassis engineering aiming at high-yield medicinal essential-oil production.

## 2. Results

### 2.1. Identification of DXS Genes in the Pan-Genome of Lauraceae

**Figure 1:**
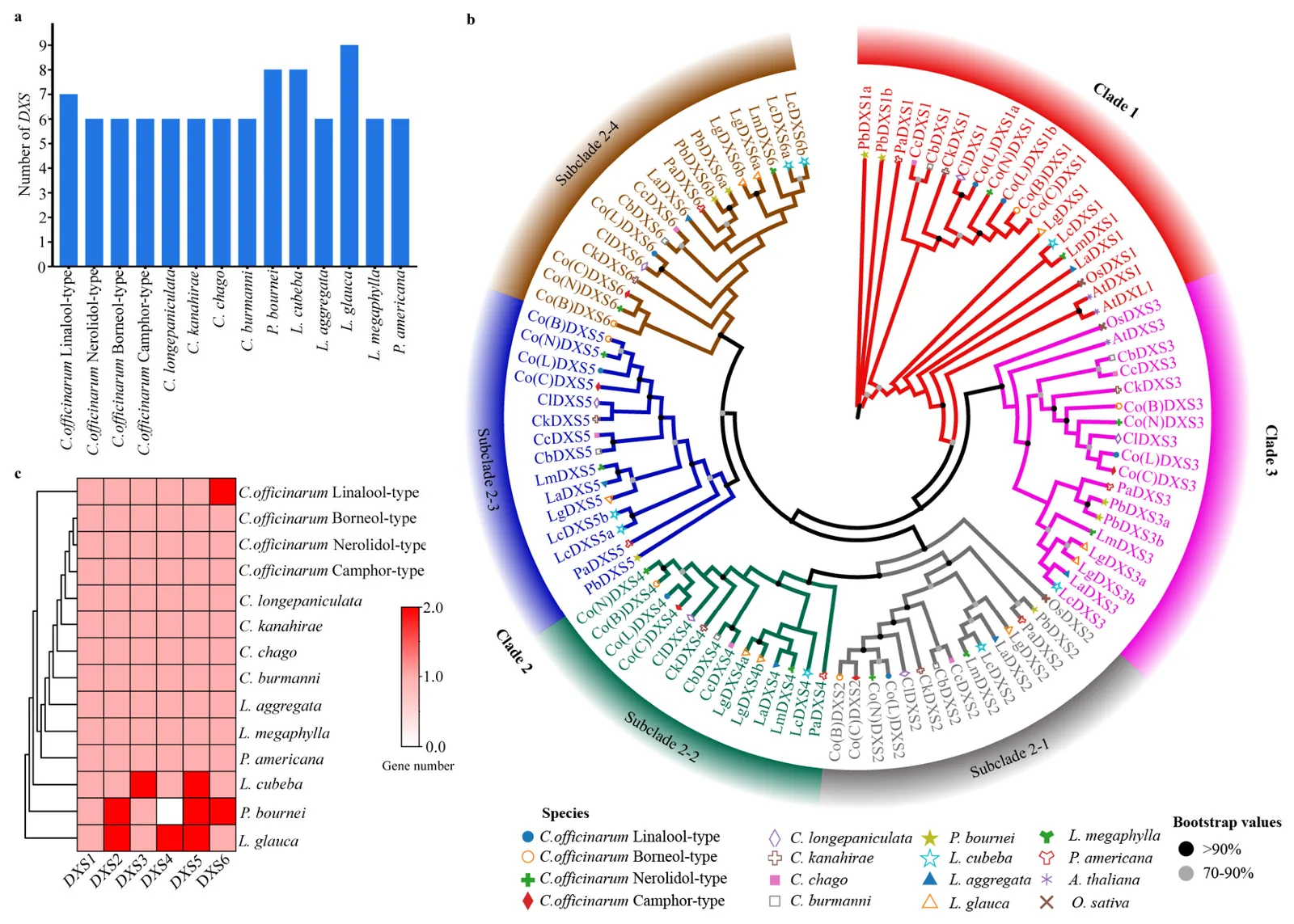
Genomic characterization of *DXS* genes across 14 Lauraceae genomes, including copy-number distribution, phylogenetic relationships, and copy-number variation (CNV) profiles of the orthogroup. (**a**) Statistics of *DXS* gene copy numbers in individual Lauraceae genomes. (**b**) Phylogenetic tree of DXS family proteins from Lauraceae species. (**c**) Gene composition and copy-number distribution of six conserved *DXS* orthogroups.

A genome-wide survey of *DXS* genes across 14 Lauraceae genomes derived from 11 species identified a total of 92 full-length *DXS* sequences. Among these candidates, nine sequences were annotated as pseudogenes due to the presence of frameshift mutations and premature stop codons (Supplementary Table S1). *L. glauca* possessed the largest *DXS* gene repertoire with nine members, whereas most Lauraceae genomes harbored six homologs (Figure 1a). Physicochemical property analysis revealed highly conserved characteristics among DXS proteins. Protein lengths ranged from 705 to 736 amino-acid residues, with molecular weights varying from 76.10 to 79.81 kilodaltons (kDa) and theoretical isoelectric points (pI) ranging from 6.04 to 8.16. Instability-index prediction indicated that 73.92% of DXS proteins were unstable in vitro, and all DXS polypeptides exhibited negative GRAVY values, suggesting universal hydrophilic properties (Supplementary Table S1). Subcellular-localization prediction further confirmed that all DXS proteins are exclusively targeted to chloroplasts, consistent with plastid-localized biosynthesis of monoterpenoids and diterpenoids in plants (Supplementary Table S1).

### 2.2. Phylogeny and Genomic Distribution

Phylogenetic analysis categorized the 92 identified Lauraceae DXS proteins into three well-supported clades (Figure 1b). Clade 1 clustered with canonical *DXS1* homologs from *A. thaliana* and *O. sativa*, suggesting its conserved function in primary terpenoid metabolism. Clade 2 contained four distinct subclades corresponding to Lauraceae *DXS2*, *DXS4*, *DXS5*, and *DXS6*, which grouped closely with the rice *DXS2* homolog and indicated potential roles in specialized secondary-metabolism regulation. Clade 3 formed an independent monophyletic lineage together with *DXS3* sequences from *A. thaliana* and *O. sativa*. This clade exhibited obvious functional divergence, and most of its paralogs had lost the canonical catalytic capacity of typical DXS enzymes [13].

Gene-distribution analysis showed that five *DXS* orthogroups, all except *DXS4,* were universally conserved across the 14 genomes (Figure 1c). *DXS4* was absent from *P. bournei*, most likely owing to a 23.5 kilobase (kb) genomic gap within the syntenic block; notably, its ortholog was recovered in an alternative assembly [45]. CNV was also observed across the family. Specifically, *DXS3* was duplicated in linalool-type *C. officinarum* and *P. bournei*; *DXS3* was duplicated in *P. bournei* and *L. glauca*; *DXS4* was duplicated exclusively in *L. glauca*; *DXS5* retained two copies in *L. cubeba*; and *DXS6* expanded in *L. cubeba*, *P. bournei*, and *L. glauca.*

### 2.3. Evolutionary Dynamics and Synteny

**Figure 2:**
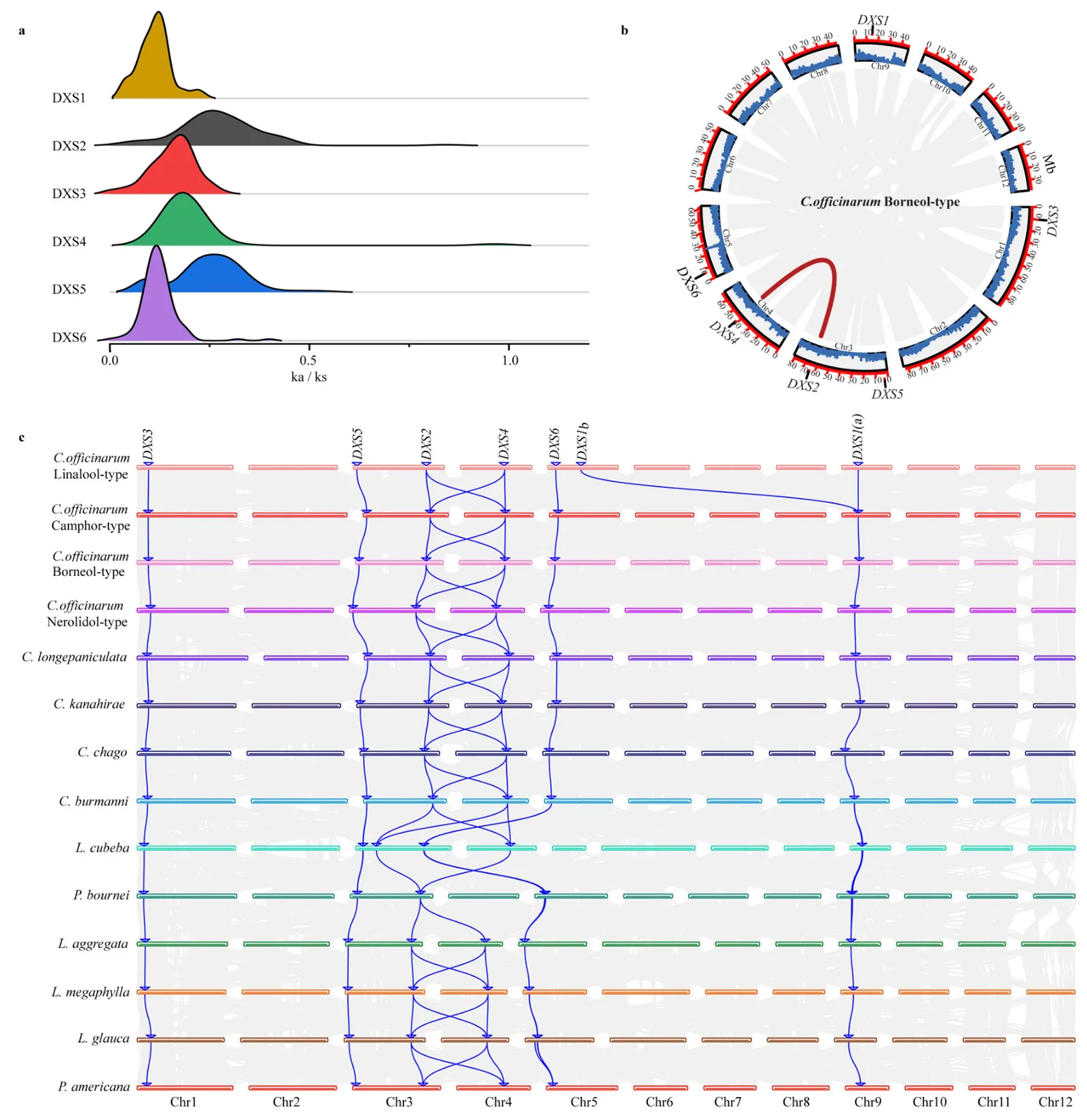
Selective-pressure and collinearity analyses of *DXS* genes across Lauraceae species. (**a**) Distribution of *Ka*/*Ks* ratios for six *DXS* orthogroups among 14 Lauraceae genomes. Median *Ka*/*Ks* values range from 0.10 to 0.40. (**b**) Circos plot showing chromosomal positions and intraspecific paralogous relationships of *DXS* loci in borneol-type *C. officinarum*. Red lines represent paralogous gene pairs of *DXS*. Grey lines represent other paralogous gene pairs within collinear blocks. (**c**) Cross-genome synteny of *DXS* loci among Lauraceae species. Blue lines represent orthologous gene pairs of *DXS* among Lauraceae genomes, and grey lines indicate other orthologous gene pairs between Lauraceae genomes.

To evaluate selective constraint and duplication history of the *DXS* family, we calculated nonsynonymous-to-synonymous substitution rate ratios (*Ka*/*Ks*) for all *DXS* genes. For all six orthogroups, *Ka*/*Ks* distributions peaked at values below 1 in every species (Figure 2a and Supplementary Table S2; median *Ka*/*Ks* approximately 0.10–0.40), indicating that DXS proteins were under strong and pervasive purifying selection across Lauraceae.

In the Circos map of the borneol-type *C. officinarum* genome (Figure 2b), 12 chromosomes were displayed with physical positions of *DXS* loci, and paralogous relationships were indicated by red links. In total, 16 intraspecific paralogous pairs were identified across the 14 Lauraceae genomes based on MCScanX-derived collinearity analysis. These pairs were located within conserved syntenic blocks and were thereby classified as WGD/SD (Figure 2b, Supplementary Figure S1, and Supplementary Table S2). Notably, these 16 paralogous pairs exhibited substantially divergent duplication ages, as reflected by their *Ks* values listed in Supplementary Table S2. An additional six gene pairs, including *LgDXS3a*–*LgDXS3b* and *LgDXS4a*–*LgDXS4b* in *L. glauca*, *PbDXS1a*–*PbDXS1b* and *PbDXS6a*–*PbDXS6b* in *P. bournei*, and *LcDXS5a*–*LcDXS5b* and *LcDXS6a*–*LcDXS6b* in *L. cubeba* were likely classified as dispersed duplicates; although they showed ~98% sequence identity, no collinear signal was detected in their flanking genomic regions.

Two lineage-specific rearrangements were detected (Figure 2c). First, *DXS6* was translocated from Chr5 to Chr3 in *L. cubeba*. Second, *DXS4* could not be identified in the *P. bournei* genome assembly used in this study. According to a previously reported alternative *P. bournei* genome assembly [45], *DXS4* is present and its flanking genes are located within conserved syntenic blocks. Therefore, the absence of *DXS4* in our employed assembly most likely stemmed from an assembly gap rather than true biological gene loss. Furthermore, partial interspecific syntenic blocks were detected surrounding *DXS1b* from linalool-type *C. officinarum* and *DXS1* orthologs from other Lauraceae species, as well as around *DXS5b* from *L. glauca* and *DXS5* orthologs from other Lauraceae species. Dense inter-species syntenic connections between *DXS2* and *DXS4* supported that they represented a paralogous pair derived from WGD or SD. By contrast, discrete syntenic blocks for the remaining *DXS* members across species corresponded to their conserved orthologous evolutionary relationships.

### 2.4. Gene Structure, Conserved Motifs, and Promoter Elements

**Figure 3:**
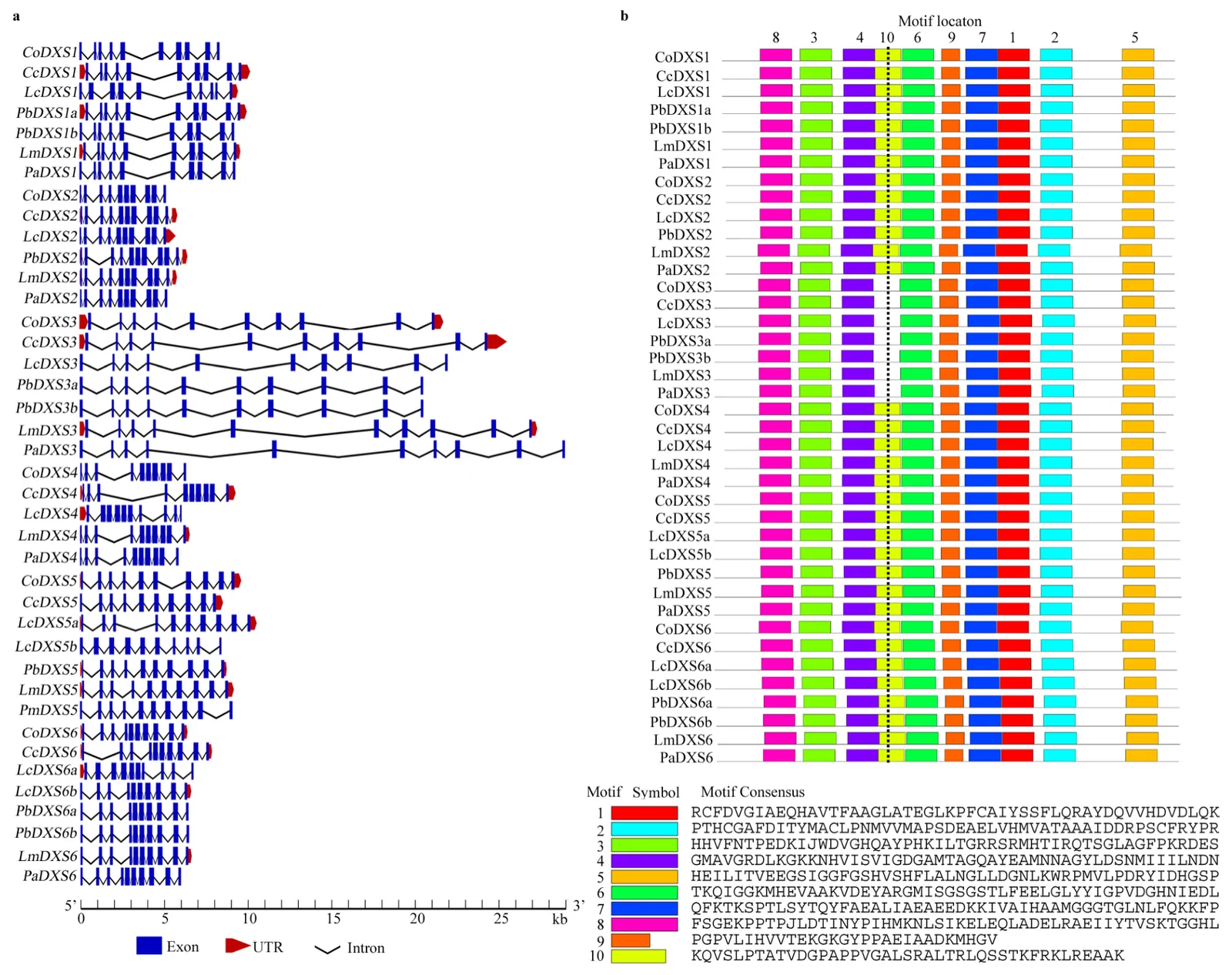
Gene structure and conserved motifs of Lauraceae DXS proteins. (**a**) Exon–intron organization of *DXS* genes. (**b**) Conserved motif patterns of DXS proteins. Dashed lines indicate the positions where motif 10 is absent, highlighting motif loss events among DXS proteins.

All *DXS* genes from Lauraceae contained ten exons, with substantial intron-length divergence among orthogroups. Given their highly conserved sequences and gene structures, representative *DXS* gene-structure diagrams and conserved protein motifs from six genera (*Camphora*, *Cinnamomum*, *Phoebe*, *Litsea*, *Lindera*, *Persea*) were selected for presentation in Figure 3. *DXS3* possessed extremely long introns, resulting in a total gene length exceeding 20 kb, whereas *DXS2*, *DXS4*, and *DXS6* showed relatively compact gene structures ranging from 6 kb to 9 kb (Figure 3a). Ten core conserved motifs were detected in the majority of DXS proteins. Notably, motif 10 was specifically absent from DXS3 members (Figure 3b). Motif 4 corresponded to the canonical TPP-binding domain, which was indispensable for catalytic activity. Motif 8 contained the first GAP (glyceraldehyde 3-phosphate)-binding motif, and motif 9 harbored the second GAP-binding motif. Motif 2 mapped to the Transketolase-C-binding site.

**Figure 4:**
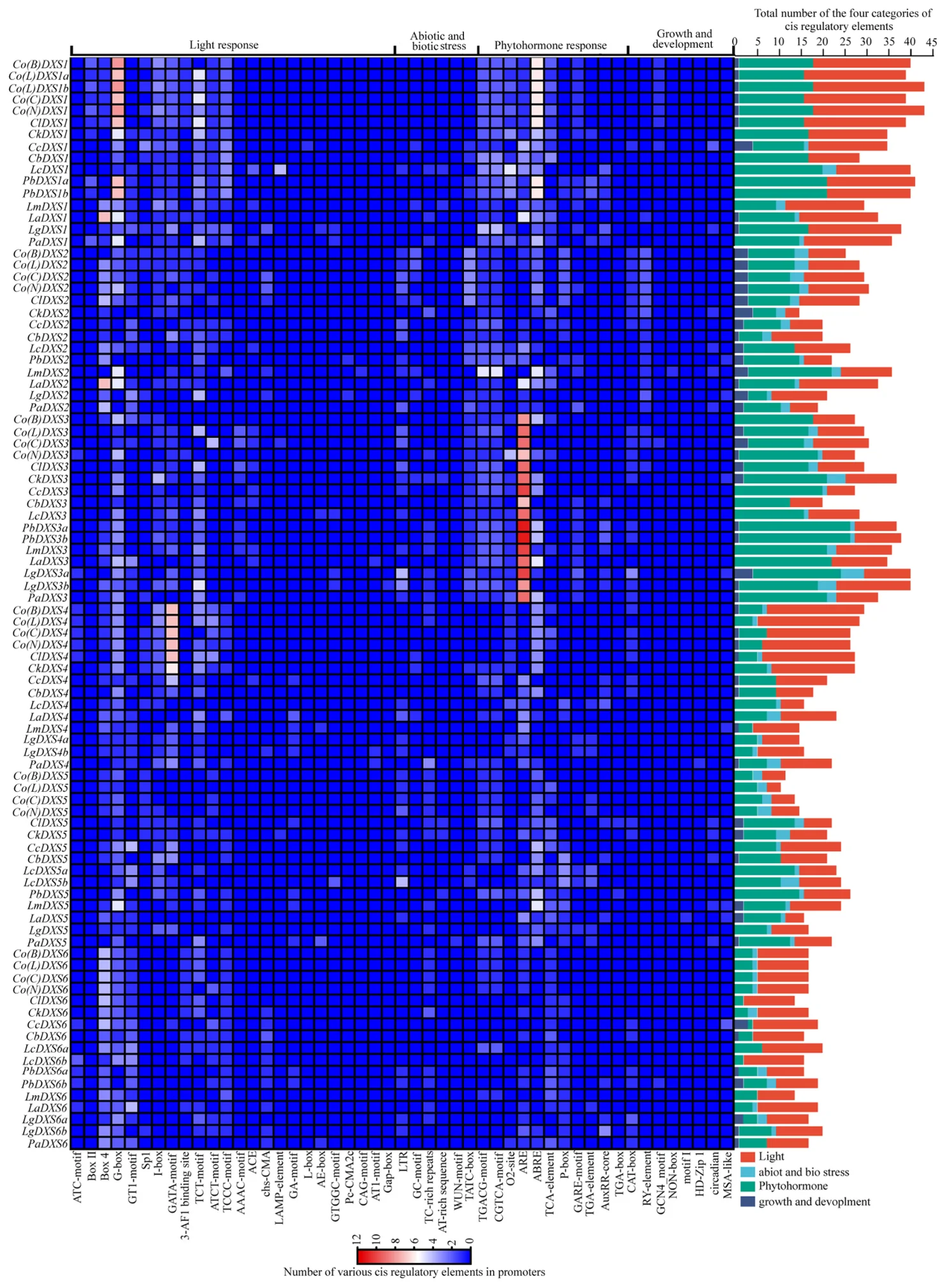
Prediction and analysis of cis-regulatory elements in the promoters of Lauraceae *DXS* genes. The heatmap shows the distribution of diverse cis-regulatory elements identified within the promoter regions of *DXS* genes. Each row represents one *DXS* gene, and each column corresponds to a specific type of cis-element. The bar plot quantifies the total number of predicted cis-regulatory elements for each *DXS* gene.

Cis-regulatory-element (CRE) profiling of 92 *DXS* promoter sequences identified 1100 light-responsive and 840 hormone-responsive elements, whose abundances exceeded those of motifs associated with abiotic stress, biotic stress, and plant development (Figure 4). Light-responsive elements showed clade-specific distribution patterns: the G-box was enriched in *DXS1* promoters, Box 4 was predominant in *DXS2* and *DXS6*, and the GT1-motif was prevalent within *DXS6*. By contrast, I-box and Sp1 elements were randomly distributed among subfamilies, with the exception of *DXS6*. Hormone-related elements also displayed obvious inter-clade variation: the gibberellin-responsive TATC-box reached its highest abundance in *DXS2*, the ABA-responsive ABRE was most enriched in *DXS1*, while the JA-associated CGTCA-motif showed no detectable clade-level bias. Overall, *DXS1* and *DXS3* harbored a greater total number of CREs, driven primarily by the accumulation of hormone- and stress-related motifs. Divergent CRE profiles were observed even for orthologs belonging to the same subclade; for instance, the *CkDXS2* promoter contained only three light-responsive elements, while *LaDXS2* harbored as many as 20. Two signature cis-elements displayed orthogroup-specific enrichment: the anaerobic-induction-related ARE was enriched in *DXS3*, and the G-box was enriched in *DXS1*. Within four chemotypes of *C. officinarum*, *DXS* promoters exhibited only minor differences in CRE composition, most notably among light-responsive elements.

### 2.5. Landscape of Structural Variations (SVs)

**Figure 5:**
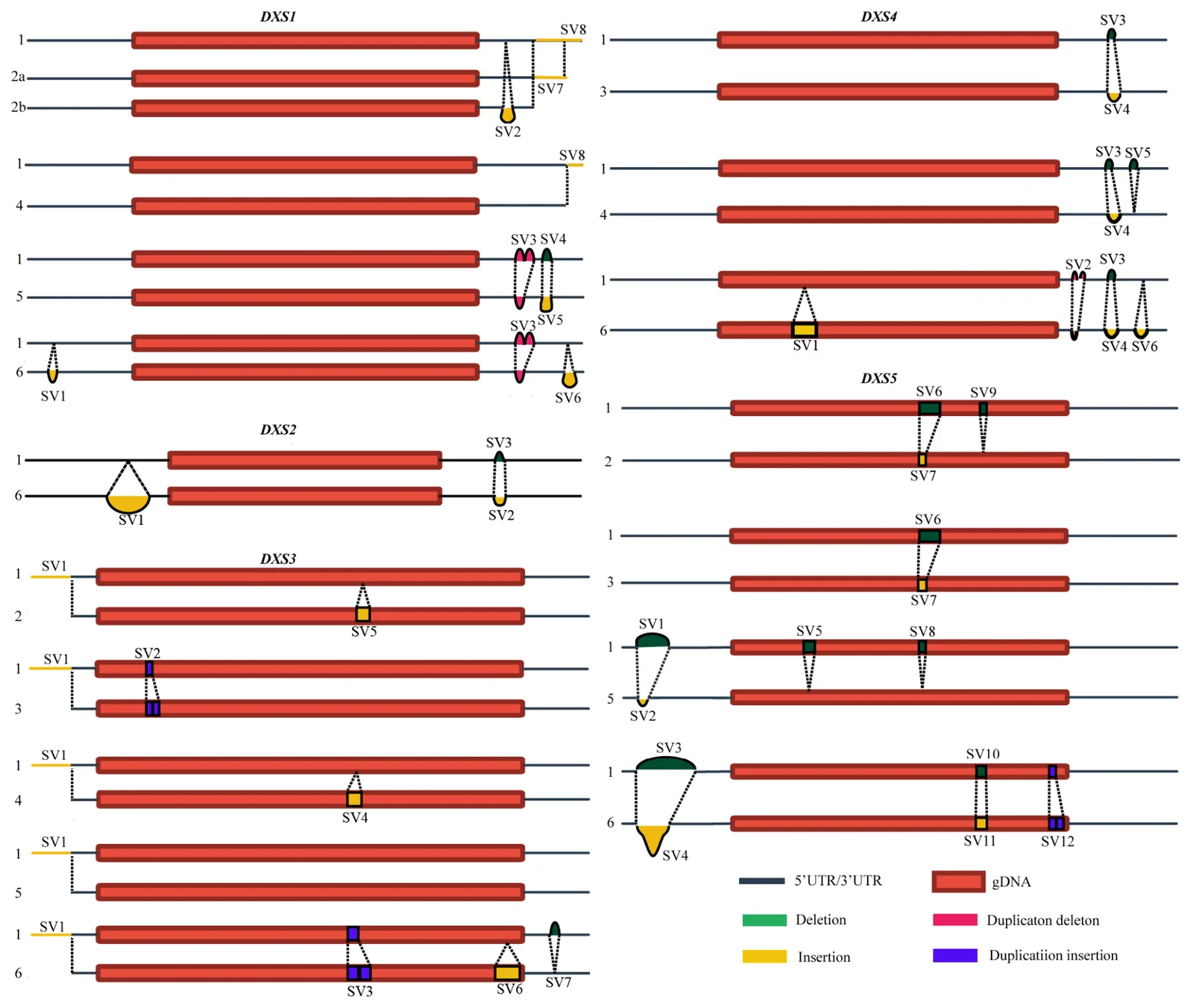
Genomic SV distribution at *DXS* loci across six genomes of the genus *Camphora*. 1. Borneol-type *C. officinarum*; 2. Linalool-type *C. officinarum*; 3. Camphor-type *C. officinarum*; 4. Nerolidol-type *C. officinarum*; 5. *C. longepaniculatum*; 6. *C. kanehirae*. Detailed information concerning SVs between borneol-type *C. officinarum* and 13 additional Lauraceae genomes is provided in Supplementary Table S3.

Comparative SV scanning based on the telomere-to-telomere (T2T) genome of borneol-type *C. officinarum*, together with 13 additional Lauraceae genomes, identified 571 high-confidence SVs across *DXS* loci (Figure 5 and Supplementary Table S3). These SVs consisted of 274 insertions, 261 deletions, 21 repeat insertions, and 15 repeat deletions, which exhibited uneven spatial distribution: 39.93% resided in intronic regions, 32.75% in the 3′ untranslated region (UTR), and 27.32% in the 5′ UTR.

Hierarchical SV divergence existed across different taxonomic levels. Inter-genus comparisons yielded higher SV counts than intra-genus comparisons within the genus *Camphora* and intra-specific comparisons among distinct *C. officinarum* chemotypes. Paralog-level quantification revealed heterogeneous SV abundance among *DXS* subfamilies. *DXS5* exhibited the highest density (8.14 SVs per locus), whereas *DXS6* exhibited the lowest SV density (4.38 SVs per locus) and possessed the most stable genomic structure.

### 2.6. Spatiotemporal Expression Patterns in C. officinarum

**Figure 6:**
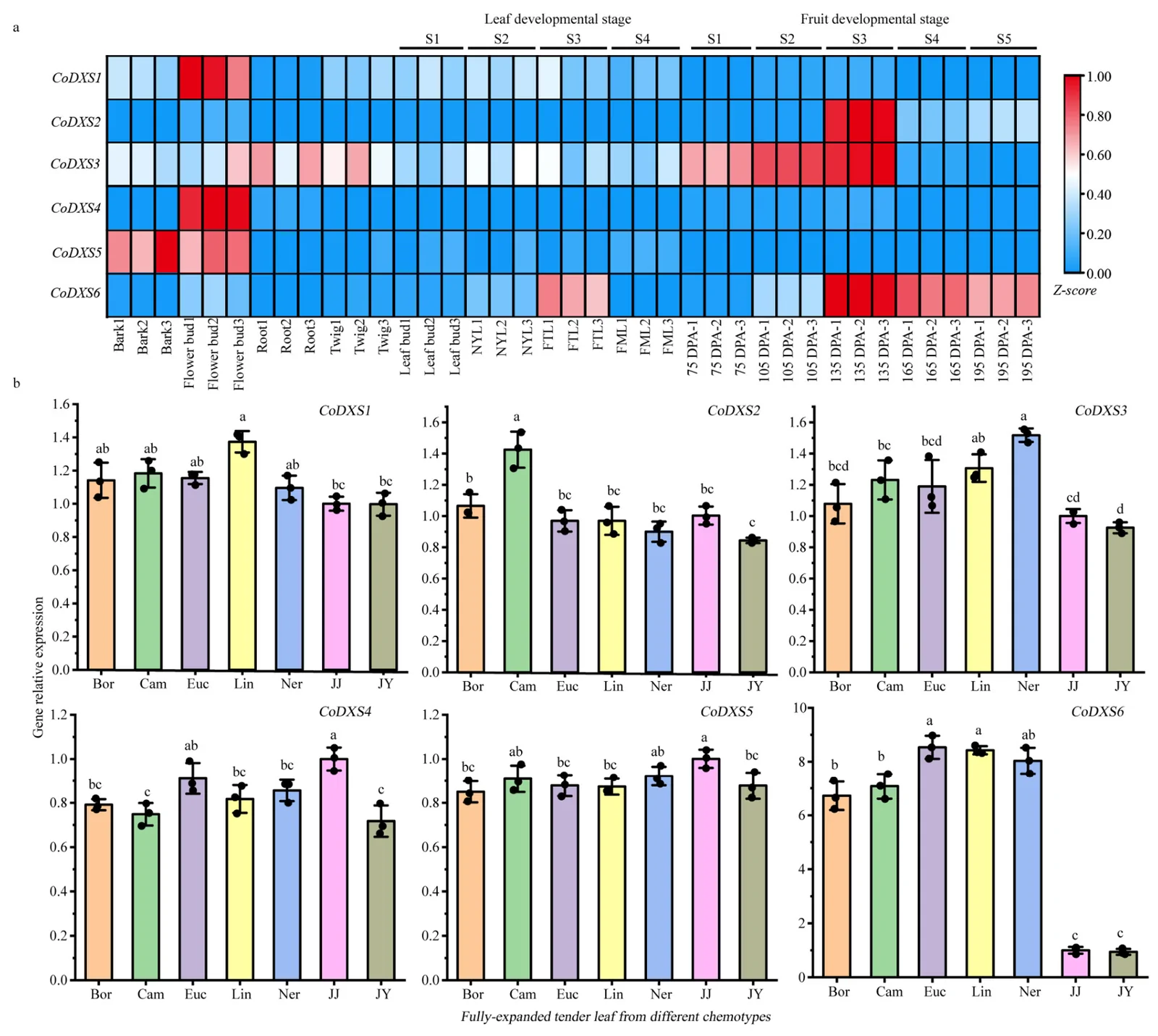
Spatiotemporal and chemotype-dependent expression patterns of *DXS* genes in *C. officinarum*. (**a**) Tissue- and developmental stage-specific *DXS* expression profiles from transcriptome -derived TPM data (triplicate). Normalized TPM values for each gene across tissues and developmental stages are provided in Supplementary Table S4. The color scale represents relative expression intensity. (**b**) qRT-PCR comparison of *DXS* expression in FTL across seven chemotypes. Bor (borneol-type), Cam (camphor-type), Euc (1,8-cineole-type), Lin (linalool-type), Ner (nerolidol-type), JJ (methyl eugenol-type), JY (methyl isoeugenol-type). Different lowercase letters denote significant differences among chemotypes by one-way ANOVA followed by Tukey’s multiple-comparison test (*p* < 0.05). Means sharing at least one common letter are not significantly different. Error bars represent mean ± standard deviation.

Transcriptome profiling across multiple tissues and developmental stages revealed distinct expression specificity among *CoDXS* paralogs (Figure 6a and Supplementary Table S4). *CoDXS2* exhibited fruit-specific activity during middle-to-late fruit development (TPM (transcripts per kilobase of exon model per million mapped reads) ≈ 10), peaking at 105 days post-anthesis (DPA; TPM > 60). This gene was nearly silent (TPM < 1) in bark, roots, twigs, and all leaf-development stages, including leaf buds, newly expanded young leaves (NYL), fully expanded tender leaves (FTL), and fully sclerified mature leaves (FML). *CoDXS3* showed constitutive but low-level expression (TPM ≈ 10), with further reduced transcript abundance in leaf buds, FML, and mature fruits (TPM ≈ 3). *CoDXS5* was weakly expressed in flower buds and bark. *CoDXS4* displayed strict, high-level flower-bud-specific expression. *CoDXS6* possessed a broader expression profile, with elevated transcript levels in flower buds, NYL, and 105-DPA fruits, and significant up-regulation in FTL and late-stage fruits (135, 165, and 195 DPA). Transcripts of *CoDXS1* were negligible or undetectable in roots and fruits harvested at 75, 105, 165, and 195 DPA, yet maintained robust constitutive expression in the remaining tissues.

Quantitative real-time PCR (qRT-PCR) analysis was performed to examine *DXS* expression in FTL samples from seven *C. officinarum* chemotypes (essential-oil profiles in Supplementary Table S5 and Supplementary Figure S2). Expanding leaves were the major site for leaf-oil biosynthesis and chemotype classification in *C. officinarum*; we therefore used these samples to screen *DXS* candidates for leaf chemotype divergence, not to assess general tissue-wide expression across Lauraceae. The results revealed significant inter-chemotype expression differences exclusively for *CoDXS6* (Figure 6b). *CoDXS6* was strongly upregulated in terpenoid-dominant chemotypes but barely detectable in phenylpropanoid-dominant chemotypes, with a maximum fold difference in expression ranging from 6.32- to 9.18-fold.

### 2.7. CoDXS6 as the Key Regulator of Leaf Monoterpenoid Biosynthesis

**Figure 7:**
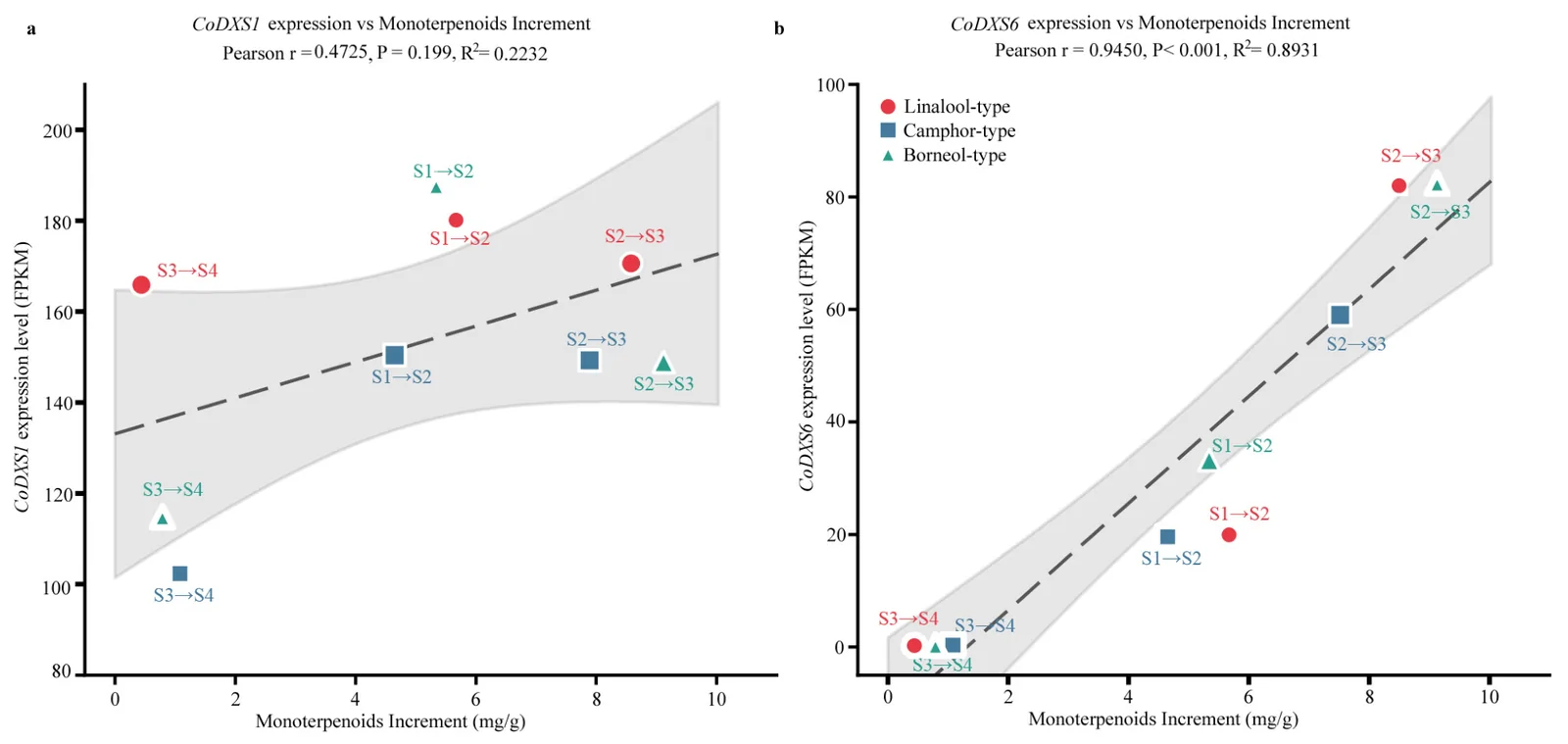
Dynamic correlation between *DXS* transcript abundance and monoterpenoid accumulation in FTL of *C. officinarum.* Dashed lines denote the linear regression fitting, and the shaded area represents the 95% confidence interval of the regression. (**a**) Transcript level of *CoDXS1* plotted against monoterpenoid increments. (**b**) Transcript level of *CoDXS6* plotted against monoterpenoid increments.

Dynamic correlation analysis between *DXS* transcript abundance and monoterpenoid accumulation across leaf developmental stages in three chemotypes (linalool-type, camphor-type, and borneol-type) identified *CoDXS6* rather than *CoDXS1* as the key regulator (Figure 7).

Monoterpenoid contents rose rapidly from the leaf bud to the NYL stage, with incremental accumulations of 5.67 ± 0.28, 4.65 ± 0.21, and 5.34 ± 0.12 mg/g in linalool-, camphor-, and borneol types, respectively. A more pronounced increase was observed from NYL to FTL, with monoterpenoid increments reaching 8.58 ± 0.32, 7.89 ± 0.41, and 9.12 ± 0.19 mg/g across the three chemotypes. Monoterpenoid biosynthesis slowed substantially at the FML stage, with only marginal content increments ranging from 0.44 ± 0.09 to 1.08 ± 0.13 mg/g. Collectively, robust monoterpenoid biosynthesis was predominantly restricted to early and middle leaf developmental stages. The estimated accumulation levels of monoterpenoids, sesquiterpenes, and phenylpropenes across seven *C. officinarum* chemotypes were calculated according to the method described in Section 4.6.

*CoDXS1* maintained constitutively high and stable expression throughout all four leaf developmental stages, and displayed no significant correlation with monoterpenoid accumulation (*r* = 0.473, *p* < 0.199) (Figure 7a). By contrast, *CoDXS6* expression increased progressively during early-to-middle leaf development, peaked at the FTL stage, and then declined sharply to a TPM value below 1.0 in FML. This dynamic expression pattern exhibited a strong positive correlation with monoterpenoid accumulation levels (*r* = 0.945, *p* < 0.001) (Figure 7b).

### 2.8. Functional Validation and Subcellular Localization

**Figure 8:**
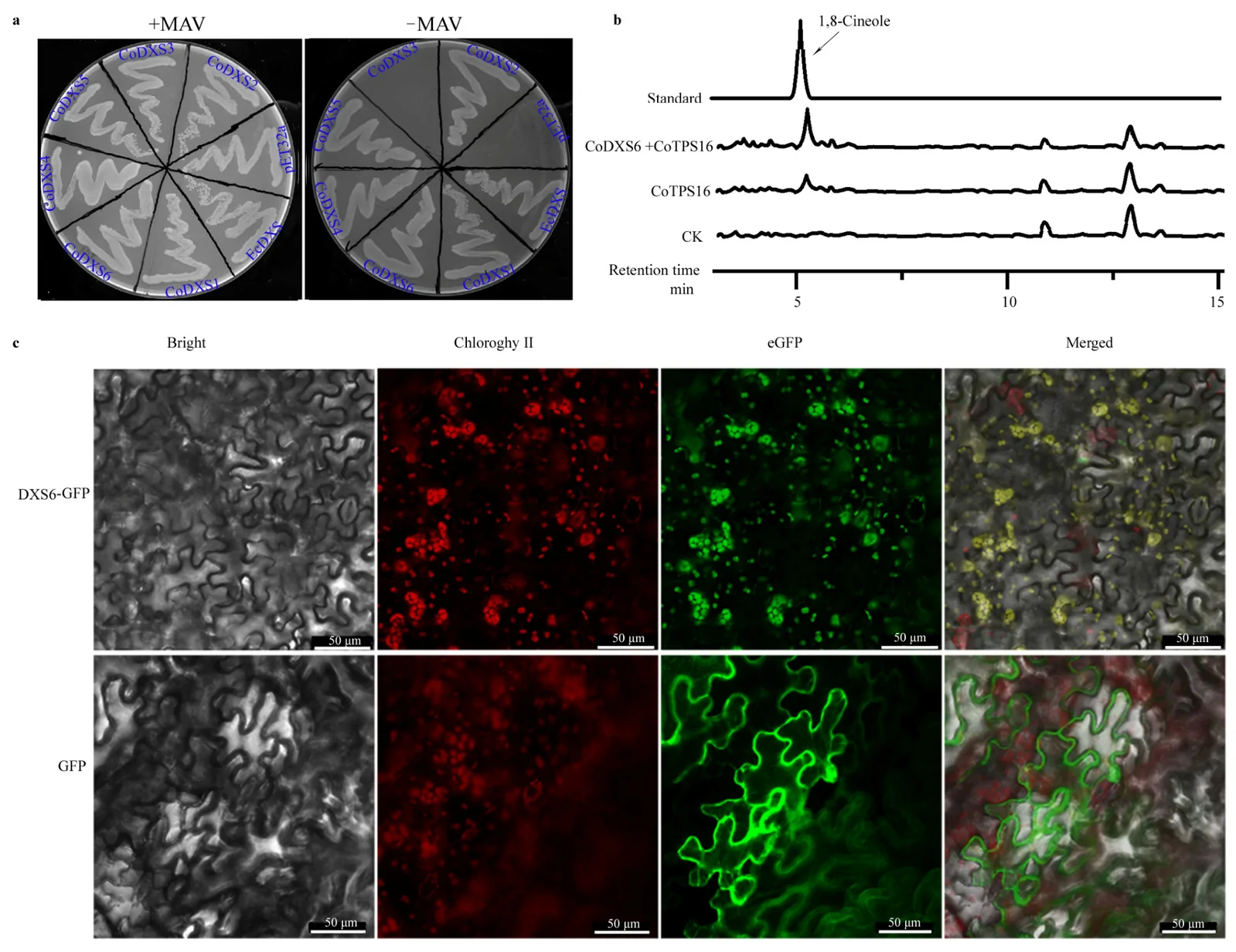
Functional validation and subcellular-localization analysis of CoDXS proteins. (**a**) In vivo functional complementation assay of CoDXS proteins using the MVA-deficient *E. coli* strain EcAB4-2, with and without exogenous MVA supplementation in the culture medium. (**b**) Transient co-overexpression assay of *CoDXS6* and *CoTPS16* in *N. benthamiana* leaves for functional characterization of CoDXS6 enzymatic activity. (**c**) Subcellular localization of the CoDXS6-eGFP fusion protein. Bright field images show cell morphology; green indicates eGFP fluorescence; red indicates chlorophyll II autofluorescence of chloroplasts; Merged represents the overlay of different channels. Scale bars = 50 μm.

All six *CoDXS* orthogroups were cloned and heterologously expressed in MVA-deficient *E. coli* strain EcAB4-2. Multiple-sequence alignment revealed that CoDXS3 lacked conserved residues at functionally critical positions, including the transketolase-pyrimidine (transket-pyr) binding site (GDGA), the C-terminal dimerization core motif (DRAG), and GAP-binding residues (H111, Y468, R496, and R554) (Supplementary Figure S3). Phenotypic complementation assays demonstrated that five CoDXS proteins, with the exception of CoDXS3, rescued the growth defect of *E. coli* strain EcAB4-2 grown on MVA-depleted medium, while empty-vector controls exhibited no rescuing phenotype (Figure 8a). These observations confirmed that five of the six CoDXS orthogroups possessed canonical in vivo DXS catalytic activity, whereas CoDXS3 was catalytically inactive.

Transient co-expression of *CoDXS6* together with the monoterpenoid synthase gene *CoTPS16* [31] in *Nicotiana benthamiana* leaves substantially elevated eucalyptol accumulation compared with plants infiltrated with *CoTPS16* alone or empty-vector controls (Figure 8b). This finding indicated that upstream precursor supply constituted a major bottleneck constraining monoterpenoid biosynthesis in planta and further validated that *CoDXS6* was functionally active in plant cells and positively modulated monoterpenoid pathway flux.

Confocal laser-scanning microscopy revealed that fluorescence from the CoDXS6-eGFP (enhanced green fluorescent protein) fusion protein overlapped exclusively with chlorophyll autofluorescence, verifying strict plastid-targeted localization (Figure 8c). Such subcellular distribution supported the notion that CoDXS6 exerted its catalytic function within the plastid-localized MEP pathway.

## 3. Discussion

### 3.1. Expansion and Evolutionary Drivers of the DXS Family in Lauraceae

DXS catalyzes the rate-limiting step of the plastidial MEP pathway and governs carbon flux toward terpenoid biosynthesis, serving as a central regulatory hub for plant growth and specialized metabolism [16]. The *DXS* gene family exhibits extensive copy-number variation across angiosperms, a pattern primarily shaped by ancient WGD events [13]. Unlike model plants that typically maintain only three *DXS* paralogs, several plant lineages have experienced moderate family expansion. In this study, we identified 92 *DXS* genes from 11 Lauraceae species, which were assigned to six well-defined orthogroups, with each genome harboring 6–9 paralogs and showing prominent interspecific copy-number divergence (Supplementary Table S1). The substantially expanded *DXS* gene repertoire endowed Lauraceae species with abundant genetic resources for functional differentiation and adaptive metabolic diversification.

Two well-characterized WGD events have occurred during Lauraceae evolution, displaying distinct genome-wide *Ks* peaks at approximately 0.83 and 0.50, corresponding to the ancient Laurales-shared WGD (~137 Mya) and the recent Lauraceae-specific WGD (~85 Mya), respectively [31,46]. These two calibrated polyploidization events provide a reliable temporal benchmark for tracing the evolutionary origin of *DXS* paralogs. Synteny analyses revealed that *DXS* family expansion was predominantly driven by WGD and SD, while no tandem duplicates were detected (Figure 2b, Supplementary Figure S1, and Supplementary Table S2). Notably, the broadly conserved *DXS2*–*DXS4* paralogous pair exhibited *Ks* values ranging from 0.433 to 0.502 (Supplementary Table S2), which precisely overlapped the genomic signature of the recent Lauraceae-specific WGD. Combined with its universal retention across Lauraceae genomes and strong purifying selection constraints, this evidence robustly confirmed that the *DXS2*–*DXS4* pair originated from the recent Lauraceae-specific WGD. Nevertheless, long-term post-WGD genomic fractionation may erode ancestral collinear signatures, and thus synteny-based evolutionary inference requires prudent interpretation [47,48].

In addition to the core paralogs originating from the recent Lauraceae-specific WGD, subsequent genome-specific duplication events further diversified the *DXS* gene pool across Lauraceae, as supported by lower *Ks* values indicative of younger duplication ages (Supplementary Table S2). Multiple genome-specific paralogous pairs, including *LgDXS6a*–*LgDXS6b* in *L. glauca*, *PbDXS3a*–*PbDXS3b* in *P. bourneii*, and *CoDXS1a*–*CoDXS1b* in *C. officinarum* (linalool type) with high sequence identity (>98%), arose from post-divergence segmental duplications, supported by collinearity evidence (Supplementary Figure S1 and Supplementary Table S2). In contrast, the dispersed duplicates identified in this study were likely generated via transposon-mediated transposition or ectopic recombination [49]. Collectively, the expansion of the Lauraceae *DXS* family constituted a continuous, multi-temporal evolutionary process: the Lauraceae-specific WGD established the foundational genetic framework, while subsequent genome-specific duplication mechanisms operated across different evolutionary timescales to enrich paralog diversity, ultimately shaping the genetic divergence and functional plasticity of the terpenoid biosynthesis pathway in Lauraceae plants.

### 3.2. Phylogenetic Architecture and Selective Constraints Differentially Shape DXS Clade Evolution in Lauraceae

DXS proteins cluster into three major clades across land plants, with mean copy numbers of 1.5 (Clade 1), 2.0 (Clade 2), and 1.2 (Clade 3) per species [13,16]. The 92 Lauraceae *DXS* sequences conformed to this broad phylogenetic framework (Figure 1b) yet exhibited prominent Clade 2-specific expansion, averaging 4.36 members per genome, more than twice the reported angiosperm average [13]. Within Clade 2, four subclades diverged from an *OsDXS2*-like ancestral node. Subclade 2-1 (*DXS2*) and subclade 2-2 (*DXS4*) formed a well-supported sister group; their status as retained ohnologs derived from the Lauraceae-specific WGD was documented in the synteny analyses (Section 3.1). By contrast, the deep evolutionary origins of *DXS5* (subclade 2-3) and *DXS6* (subclade 2-4) remained ambiguous, despite the universal retention of both subclades across Lauraceae genomes. Within these two subclades, genome-restricted duplications occurred independently in multiple species after the Lauraceae-specific WGD. For instance, *LgDXS6a*/*6b* in *L. glauca* represented a bona fide segmental-duplication-derived pair supported by collinearity, whereas high-identity copies such as *LcDXS5a*/*5b* in *L. cubeba* were classified as likely dispersed duplicates, for which the duplication mechanism could not be unambiguously resolved by current synteny data. Collectively, these genome-restricted events further augmented paralog diversity superimposed on the ancestral WGD-established genomic background.

Differential selective constraints closely mirrored this phylogenetic heterogeneity. *Ka*/*Ks* analyses showed that all three clades evolve under purifying selection (*Ka*/*Ks* < 1), yet with divergent selective intensities. Clade 1 (*DXS1*) was subjected to the strongest functional constraint (*Ka*/*Ks* = 0.12), consistent with its housekeeping role of supplying DXP for core plastidial isoprenoid metabolites required for plant growth and development [50]. Clade 3 (*DXS3*) exhibited relaxed purifying selection (*Ka*/*Ks* = 0.19), matching its documented loss of catalytic competence (Section 2.8). This functional decay was further supported by motif analysis: Clade 3 proteins possessed only nine conserved motifs and lacked motif 10. Within Clade 2, subclades displayed significantly differentiated *Ka*/*Ks* distributions (Figure 2a), pointing to variable functional constraints that might underpin the spatiotemporal divergence of terpenoid metabolism across Lauraceae tissues and developmental stages.

Genomic contextual features further corroborate coding-sequence-level conservation. Intergenomic synteny revealed that *DXS* loci maintained largely conserved chromosomal positions across the 14 surveyed genomes, indicating that this pan-family genomic arrangement was already established prior to Lauraceae diversification. *L*. *cubeba* constituted the only conspicuous exception: *LcDXS6* was located on chr3 instead of the otherwise conserved chr 5. Whether this positional shift arose from true chromosomal rearrangement, assembly artifact, or gene-annotation error awaits further experimental validation. Furthermore, conserved gene structure (all 92 genes contain 10 exons) and uniform motif composition (10 conserved motifs present in all putatively functional paralogs of Clade 1 and Clade 2) provided additional evidence for strong structural constraints acting on the DXS catalytic core.

### 3.3. SV, Cis-Regulatory Element, and Expression Profiling of DXS Genes

SVs are key genetic drivers of phenotypic differentiation, metabolic diversification, and adaptive evolution in plants [51]. Here, we utilized the T2T genome of *C. officinarum* (borneol-type) as a reference to systematically characterize SVs within *DXS* genes across 13 additional Lauraceae genomes. Our analyses showed that *DXS*-associated SVs were exclusively enriched in UTRs and introns, whereas no SVs were detected in coding sequences (Figure 5 and Supplementary Table S3). The absence of coding-region variation indicated strong purifying selection acting on *DXS* loci, consistent with its essential function as a dosage-sensitive and rate-limiting enzyme in the MEP pathway [52,53]. In contrast, non-coding regulatory regions tolerated extensive genetic variation, which might contribute to gene expression divergence.

Integrated SVs and transcript profiling revealed variable genotype–expression relationships among *DXS* paralogs across four chemotypes of *C. officinarum*. Although non-coding SVs were detected in *CoDXS1*, *CoDXS4*, and *CoDXS5*, these variants were not associated with detectable expression differences among chemotypes. This observation might indicate that these SVs resided outside functionally critical transcriptional regulatory regions. Unlike *CoDXS1*/*4*/*5*, *CoDXS3* exhibited weak chemotype-biased expression between borneol- and nerolidol-type plants, a pattern that might be linked to its regulatory SVs, albeit with a fold change below the 2-fold threshold (Figure 6b). Most prominently, *CoDXS6* displayed extreme chemotype-specific expression (6.32- to 9.18-fold) between terpenoid-rich and phenylpropene-rich chemotypes, representing the strongest divergent expression pattern among all *DXS* paralogs. This substantial expression difference raised the possibility that lineage-specific non-coding SVs contributed to the transcriptional regulation of *CoDXS6*. However, this hypothesis could not be rigorously validated at present due to the lack of high-quality genomic resources for phenylpropene-type *C. officinarum*. Future integration of genomic sequencing of phenylpropene-type accessions and CRISPR-Cas9-mediated editing of target SV loci will help establish a causal relationship between regulatory SVs and chemotype-dependent *CoDXS6* expression.

Furthermore, SV accumulation in *DXS* genes followed a clear phylogenetic distance-dependent pattern (Figure 1b and Supplementary Table S3). Closely related Lauraceae species exhibited highly conserved *DXS* genomic architectures, while phylogenetically divergent lineages accumulated substantial lineage-specific SVs, consistent with a neutral model of sequence evolution. Promoter annotation also revealed considerable qualitative and quantitative divergence in CRE composition among *DXS* homologs from different Lauraceae species (Figure 4 and Supplementary Table S3). Given the preferential localization of SVs within CRE-bearing non-coding regions, the observed interspecific CRE divergence was likely attributable, at least partially, to lineage-specific SV accumulation that remodeled regulatory sequences. Such SV-derived CRE variations provided a plausible genomic basis for functional differentiation of *DXS* genes across the Lauraceae family.

### 3.4. Identification of CoDXS6 as the Core Regulator of Leaf Monoterpenoid Biosynthesis in C. officinarum

Functional complementation assays using the MVA-deficient *E. coli* strain EcAB4-2 were applied to assess the catalytic activity of Lauraceae DXS proteins. Five Lauraceae DXS paralogs retained full catalytic capacity and could functionally rescue the defective MEP-pathway phenotype of this mutant strain. By contrast, DXS3 (Clade 3) lost canonical DXS catalytic function (Figure 8a). Notably, catalytic inactivation of Clade 3 showed scattered phylogenetic distribution across angiosperms: loss-of-function was documented in *A. thaliana* [13] and *Solanum lycopersicum* [54], while functional Clade 3 homologs existed in *Monarda citriodora* [17] and *Aquilaria sinensis* [55]. Such inconsistent functional status indicated independent functional divergence of Clade 3 DXS during angiosperm evolution.

In CoDXS3, the loss of catalysis arose from widespread degeneration of essential residues required for canonical DXS activity (Supplementary Figure S3). Disruptions within the transket-pyr domain (GDGA motif for TPP cofactor and substrate recognition), the C-terminal DRAG dimerization motif, as well as key GAP-binding residues collectively impaired cofactor anchoring, pocket integrity, homodimer assembly and substrate docking [56,57]. Cross-species protein alignment further demonstrated that these deleterious substitutions were fixed in DXS3 orthologs across multiple Lauraceae species, indicating that catalytic inactivation represented a clade-conserved trait of Lauraceae DXS3, rather than a sporadic mutation restricted to *C. officinarum*.

Multiple lines of functional evidence supported the positive regulatory roles of Lauraceae *DXS* paralogs in terpenoid biosynthesis. Transient co-expression of *CoDXS6* and monoterpene synthase gene *CoTPS16* [31] in *N. benthamiana* leaves markedly increased monoterpenoid accumulation, with 1,8-cineole as the dominant product (Figure 7b). These results were consistent with earlier functional reports in Lauraceae. Previous work showed that transient overexpression of *LcDXS2*, *LcDXS6a* and *LcDXS6b* (formerly annotated as *LcDXS3-5*) in *L. cubeba* elevated mono- and sesquiterpenoid levels [38]; heterologous overexpression of *CbDXS1* in *Arabidopsis* also enhanced DXS activity and overall terpenoid metabolic flux [44]. Collectively, Clade 2 members such as *DXS6* predominantly modulated secondary terpenoid metabolism, whereas the Clade 1 paralog *DXS1* sustained primary housekeeping isoprenoid biosynthesis.

Tissue-specific expression patterns further reinforced this functional divergence (Figure 6a). *CoDXS1* (Clade 1) was constitutively expressed across bark, twigs, flower buds and leaves, matching its basal housekeeping function. *CoDXS3* (Clade 3) exhibited universally low transcript abundance, consistent with its loss of catalytic activity. Clade 2 paralogs displayed pronounced tissue-expression divergence: *CoDXS6* was highly expressed in developing leaves and fruits; *CoDXS2* was fruit-development-specific. This fruit-preference was phylogenetically conserved in *L. cubeba*, where its corresponding orthologs were predominantly expressed in pericarp and were subject to regulation by LcMYB43 [28,38]. Additionally, *CoDXS4* showed flower-bud-specific expression, while *CoDXS5* remained transcriptionally silent across examined tissues.

Correlation analyses identified *CoDXS6* as a core regulator of leaf monoterpenoid biosynthesis. Although both *CoDXS1* and *CoDXS6* were highly expressed during leaf development (Figure 6a), only *CoDXS6* transcript abundance correlated positively with monoterpenoid accumulation (Figure 7). Its expression specificity, phylogenetic placement within Clade 2, intact protein structure and proven enzymatic activity collectively designated *CoDXS6* as the major gene channeling carbon flux toward leaf monoterpenoids. Furthermore, *CoDXS6* was strongly upregulated in terpenoid-rich compared with phenylpropene-rich chemotypes (Figure 6b), suggesting its transcriptional variation contributed to chemotype differentiation. Given that leaf essential oils of multiple *C. officinarum* chemotypes are dominated by monoterpenoids [31], *CoDXS6* constitutes a promising target for metabolic engineering to improve essential-oil yield.

## 4. Materials and Methods

### 4.1. Genome Data and Plant Materials

Genomic data of 11 Lauraceae species were obtained from public databases and personal communications, including *C. officinarum* (linalool-, camphor-, and nerolidol-type) [30,31,32], *C. longepaniculatum* (1,8-cineole-type) [33], *C. kanehirae* [34], *C. chago* [35], *C. burmannii* [36], *P. bournei* [37], *L. cubeba* [38], *L. aggregata* [39], *L. glauca* [40], *L. megaphylla* [41], and *P. americana* [42].

Leaf samples of seven *C. officinarum* chemotypes (linalool-, 1,8-cineole-, camphor-, borneol-, nerolidol-, methyl eugenol-, and methyl isoeugenol-type) were collected from the Jiangxi Academy of Forestry. For each chemotype, 30–100 g of fresh leaf tissue was harvested for essential oil extraction and chemical composition analysis. Approximately 1 g of FTL was immediately snap-frozen in liquid nitrogen, ground to fine powder, and stored at −80 °C for total RNA isolation. The obtained RNA samples were subsequently used for qRT-PCR assays and gene cloning.

### 4.2. Bioinformatics Analysis of DXS Genes in 14 Lauraceae Genomes

The hidden Markov model (HMM) profile of the DXS domain (PF13292) was downloaded from the InterPro database (https://www.ebi.ac.uk/interpro/ (accessed on 6 July 2026)). Three *A. thaliana DXS*-related genes, including *AtDXS1* (GenBank accession: CAB78598.1), *AtDXL1* (AEE76516.1), and *AtDXS3* (CAB96673.1), were adopted as reference queries for homologous gene screening. Candidate DXS homologs were mined from 14 Lauraceae proteomes via BLASTP [58] and HMMER v3.3.2 (http://hmmer.org/ (accessed on 8 July 2026)), with an E-value cutoff of 1 × 10^−10^. To reduce false positives caused by genome sequencing, assembly, and annotation artifacts, all candidate sequences were verified against their corresponding genomic and transcriptomic datasets. Full-length *DXS* sequences were manually curated, while redundant, truncated, and error-prone sequences were excluded from subsequent analyses.

Physicochemical properties of DXS proteins, including amino-acid length, molecular weight, theoretical pI, instability index, and grand average of hydropathicity (GRAVY), were calculated using ExPASy ProtParam (https://web.expasy.org/protparam/ (accessed on 10 July 2026)). Subcellular localization was predicted with WoLF PSORT (https://wolfpsort.hgc.jp/ (accessed on 10 July 2026)). Multiple-sequence alignment of DXS proteins was performed employing the ClustalW algorithm (version 2.0), and conserved motifs and variable regions were visualized via GeneDoc v2.7 (https://genedoc.software.informer.com/ (accessed on 12 July 2026)).

### 4.3. Phylogenetic, Collinearity, Ka/Ks, and SV Analyses

A total of 92 DXS protein sequences from 14 Lauraceae genomes, together with homologs from *A. thaliana* and *O. sativa* (serving as outgroups), were aligned using MAFFT v7.490 [59]. Low-quality aligned regions and gaps were subsequently trimmed with trimAl v1.4.1 [60] to generate a high-confidence alignment matrix. A maximum likelihood (ML) phylogenetic tree was constructed using IQ-TREE v2.1.2 [61], with the optimal substitution model automatically determined via the built-in ModelFinder module [62]. Branch support was evaluated based on 1000 ultrafast bootstrap replicates. The final tree was visualized and refined using ChiPlot 2.6.1 (https://www.chiplot.online/tvbot.html (accessed on 15 July 2026)).

Based on the phylogenetic clade classification, the CNV of *DXS* genes across different evolutionary clades was statistically summarized for the Lauraceae genomes, with CNV defined as a gene copy number ≥ 2. To elucidate the molecular mechanisms driving *DXS* gene expansion and verify phylogenetic tree topological reliability, intragenomic collinearity analysis was performed using MCScanX [63] to identify WGD or SD, TD, and DD events. Intergenomic collinearity analysis was further conducted to evaluate the chromosomal positional conservation of *DXS* loci among Lauraceae species.

To assess the selective pressure acting on the *DXS* gene family, orthologous gene pairs were aligned, and their *Ka*/*Ks* ratios were calculated using KaKs_Calculator 2.0 [64]. Gene pairs with *Ks* values greater than 2 were discarded to eliminate the interference of sequence substitution saturation. The *Ka*/*Ks* ratios were interpreted following conventional evolutionary criteria: *Ka*/*Ks* < 1 indicates purifying selection, *Ka*/*Ks* ≈ 1 indicates neutral evolution, and *Ka*/*Ks* > 1 indicates positive selection.

To characterize the SV patterns, full-length genomic sequences of *DXS* genes and their 3 kb upstream and downstream flanking sequences were extracted from 14 Lauraceae genomes using TBtools-II v2.486 [65]. The annotated *DXS* sequences from the high-quality T2T genome of borneol-type *C. officinarum* were used as the references for homologous comparison via BLASTN [58]. SVs were identified under rigorous thresholds, including a minimum variant length of 50 bp and an alignment quality score of no less than 20. Custom Python 3.9.12 scripts were adopted to statistically analyze SV distribution in different functional regions of *DXS* genes (5′ UTR, intron, and 3′ UTR).

### 4.4. Gene Structure, Conserved Motif, and Promoter Cis-Element Analysis

The gene structural features (exon–intron architecture) of Lauraceae *DXS* genes were analyzed and visualized using the Gene Structure Display Server 2.0 (GSDS 2.0; http://gsds.gao-lab.org/ (accessed on 15 July 2026)) using genomic GFF annotation files. Conserved protein motifs were predicted with the MEME Suite (https://meme-suite.org/meme/ (accessed on 15 July 2026)), where the maximum number of motifs was set to 10. For cis-element identification, 2 kb sequences upstream of the translation start site of each *DXS* gene were extracted and regarded as promoter regions. Putative cis-acting regulatory elements were predicted and annotated against the PlantCARE database (https://bioinformatics.psb.ugent.be/webtools/plantcare/html/ (accessed on 16 July 2026)). The detected cis-elements were further categorized based on their functional annotations to uncover the potential transcriptional regulatory mechanisms contributing to functional differentiation of *DXS* genes in Lauraceae.

### 4.5. Expression Profiling and Quantitative Analysis

Transcriptome-based expression profiling was conducted using multi-omics datasets of *C. officinarum* to systematically characterize the tissue-specific and developmental expression patterns of *CoDXS1*–*CoDXS6*. The tested samples encompassed diverse tissues (roots, stems, twigs, bark, and flower buds), four leaf developmental stages of linalool-type [leaf bud (S1), NYL (S2), FTL (S3), and FML (S4)], and five fruit developmental stages at 75 (S1), 105 (S2), 135 (S3), 165 (S4), and 195 (S5) DPA. All samples were collected with three independent biological replicates. Transcript abundance (TPM values) of *CoDXS1*–*CoDXS6* was extracted from transcriptome datasets for subsequent analysis.

To remove expression bias among samples, raw TPM values were subjected to log_2_-transformation (TPM + 1) to mitigate the effects of extreme values, followed by Z-score normalization. A heatmap was constructed based on the normalized expression matrix to illustrate the differential expression profiles of *CoDXS1*–*CoDXS6* across tissues and developmental stages.

qRT-PCR was performed to quantify the transcript levels of *DXS* genes in FTL of seven *C. officinarum* chemotypes. Gene-specific primer sequences were listed in Supplementary Table S6. The *C. officinarum β*-actin gene was used as the internal reference. qRT-PCR amplification was conducted with TB Green^®^ Premix Ex Taq™ II (Tli RNaseH Plus) (Takara Biomedical Technology (Beijing) Co., Ltd., Beijing, China) in a 20 μL reaction system, containing 10 μL 2 × TB Green Premix, 0.5 μL forward primer (10 μM), 0.5 μL reverse primer (10 μM), and 100 ng cDNA template, with nuclease-free ddH_2_O supplemented to a final volume of 20 μL. Amplification and melt-curve analysis were carried out on a Bio-Rad CFX96 real-time PCR system (Bio-Rad Laboratories, Inc., Hercules, CA, USA). The thermal cycling program consisted of an initial pre-denaturation step at 95 °C for 2 min, followed by 40 cycles of 95 °C for 5 s and 60 °C for 30 s. Melt-curve analysis was performed after amplification to verify primer specificity.

Relative gene expression levels were computed using the 2^−ΔΔCt^ method [66], with the methyl eugenol-type set as the control group. Three biological replicates, each derived from an independent individual plant, were included for each chemotype. One-way ANOVA followed by Tukey’s HSD post hoc test was used to assess the significance of expression differences across chemotypes. Genes exhibiting an absolute fold change ≥ 2 and *p* < 0.05 in pairwise comparisons were regarded as differentially expressed among chemotypes.

### 4.6. Determination of Leaf Essential Oil Components and Correlation Analysis

Fresh leaf samples with three independent biological replicates were sealed and kept under low-temperature conditions immediately after harvesting. Essential oil was isolated by steam distillation for 2 h using a Clevenger-type apparatus. After distillation, the obtained essential oil was weighed and stored hermetically at low temperature. The essential-oil yield (weight/weight, *w*/*w*) for each sample was calculated as the ratio of essential-oil weight to fresh-leaf weight.

Gas chromatography–mass spectrometry (GC-MS) analysis was carried out on a Shimadzu QP2020 instrument (Shimadzu Corporation, Kyoto, Japan) equipped with an SH-RXI-5SILMS capillary column (30 m × 0.25 mm × 0.25 μm). Essential oil samples were dissolved in ethanol at a concentration of 30 mg/mL for injection; the injection volume was 1.0 μL with a split ratio of 20:1. The injector temperature was set to 280 °C. The oven temperature program was as follows: held at 80 °C for 2 min, increased to 160 °C at 8 °C min^−1^, further ramped to 250 °C at 8 °C min^−1^, and maintained at 250 °C for 2 min. The EI ion-source temperature was 230 °C and the transfer-line temperature was 200 °C. Mass spectra were acquired over the *m*/*z* scan range of 50–650.

Compound identification was performed by comparing mass spectra against the NIST 8.0 mass-spectral library. Retention indices were calculated based on a series of n-alkanes (C9–C33) run under identical GC-MS conditions and further cross-referenced against published literature and NIST database records. For relative quantification, external calibration curves of reference standards (β-pinene, eucalyptol, 2-dodecanone, eugenol) were constructed using six concentration levels, and the relative percentage of each constituent in essential oil was calculated according to the reported procedure [67]. Based on the relative content data obtained from GC-MS, summed relative proportions of monoterpenoids, sesquiterpenes, and phenylpropenes per sample were multiplied by the corresponding essential-oil yield to estimate their accumulation levels in fresh leaves.

To elucidate dynamic accumulation patterns of key terpenoid metabolites during leaf development, leaves from three dominant *C. officinarum* chemotypes (linalool-, camphor-, and borneol-type) at four developmental stages (S1–S4) were analyzed for monoterpenoid content. Changes in monoterpenoid accumulation between adjacent developmental stages (S1 → S2, S2 → S3, and S3 → S4) were calculated. Combined with TPM values of *CoDXS5* and *CoDXS6* at corresponding stages (S2–S4), Pearson correlation analysis was performed to clarify the relationship between the expression of key *DXS* genes and dynamic monoterpenoid accumulation.

### 4.7. Functional Characterization and Subcellular Localization

Gene-specific primers were designed based on the *C. officinarum* genome reference sequence (Supplementary Table S6). Using cDNA from linalool-type *C. officinarum* leaf tissue as a template, target fragments were amplified with TransStart^®^ FastPfu DNA Polymerase (TransGen Biotech, Beijing, China). The thermal cycling program consisted of an initial pre-denaturation at 98 °C for 1 min, 35 cycles (98 °C for 10 s, 55 °C for 5 s, 72 °C for 1 min), and a final extension at 72 °C for 5 min. Amplified products were excised from agarose gels, purified, and cloned into the pEASY^®^-Blunt vector (TransGen Biotech). Positive clones were confirmed via Sanger sequencing.

To characterize the six CoDXS isoforms, N-terminal chloroplast transit peptides were predicted using TargetP 2.0 (https://services.healthtech.dtu.dk/service.php?TargetP-2.0 (accessed on 15 July 2026)). Coding sequences lacking the predicted transit peptides were cloned into pET-32a between EcoRI and SalI sites using Seamless Cloning Master Mix (Sangon Biotech, Shanghai, China); primers are listed in Supplementary Table S6.

The recombinant constructs *CoDXS*-pET32a and *EcDXS*-pET32a (positive control), together with empty pET-32a (negative control), were transformed into the DXS-deficient *E. coli* mutant strain EcAB4-2 [68]. This mutant carries a synthetic MVA operon that restores endogenous IPP and DMAPP, and thus viability, when exogenous MVA (Sigma-Aldrich, Inc., St. Louis, MO, USA) is supplied. In contrast, heterologous expression of a functional *DXS* restores growth on MVA-free Luria–Bertani (LB) medium. For complementation assays, transformants were streaked onto LB agar containing 50 μg/mL kanamycin and 100 μg/mL ampicillin, with or without 1 mM MVA. Plates were incubated at 37 °C overnight, and bacterial growth was recorded to evaluate the catalytic activity of each CoDXS protein.

To characterize *CoDXS6* function in planta, transient expression assays were performed in *N. benthamiana*. The full-length open reading frames (ORFs) of *CoDXS6* and *CoTPS16* (GenBank: OM721572) were cloned into pCAMBIA1300-Flag/C using the In-Fusion Cloning Kit EXV06 (Biogel GeneTech, Hangzhou, China), generating pCAMBIA1300-*CoDXS6*-Flag and pCAMBIA1300-*CoTPS16*-Flag. The constructs were introduced into *Agrobacterium tumefaciens* strain GV3101 by the freeze–thaw method. Positive transformants were cultured on LB agar medium containing 25 μg/mL rifampicin, 25 μg/mL gentamicin, and 50 μg/mL kanamycin at 28 °C for 3 days, followed by overnight incubation in 20 mL liquid LB medium at 28 °C. Cells were collected, washed, and resuspended in infiltration buffer (10 mM MgCl_2_, 10 mM 2-(N-morpholino) ethanesulfonic acid, pH 5.6, 150 μM acetosyringone) to an OD_600_ of 0.8, and incubated in the dark at room temperature for 3 h before infiltration. Three treatments were prepared: empty vector control, *CoTPS16* single expression, and *CoDXS6*/*CoTPS16* co-expression. *Agrobacterium* suspensions were infiltrated into the abaxial epidermis of leaves from six-week-old *N. benthamiana* seedlings. At 96 h post-infiltration, leaf disks were sampled, snap-frozen in liquid nitrogen, and ground to a fine powder. Three independent biological replicates were performed for each treatment. For each sample, 0.10 g of frozen powdered leaf tissue was transferred into a 20 mL headspace vial. Volatiles were collected via headspace solid-phase microextraction (HS-SPME) using a 50/30 μm DVB/CAR/PDMS fiber. The vial was incubated at 55 °C for 25 min with constant agitation for volatile absorption. Afterwards, the SPME fiber was transferred to the GC injector and desorbed at 250 °C for 5 min under splitless mode. Volatile compounds were analyzed by GC-MS adopting identical chromatographic and mass-spectrometric parameters as described in Section 4.6. Compound identification was performed by matching retention indices and mass spectra against authentic standards and/or the NIST mass-spectral library. Relative contents were calculated by peak-area normalization as documented in Section 4.6.

For subcellular localization, the fusion vector pCAMBIA1300-*GFP*-*CoDXS*6 was constructed using In-Fusion Cloning Kit EXV08 (Biogel GeneTech, Hangzhou, China). *Agrobacterium*-mediated transformation and tobacco leaf infiltration were performed as described above. At 72 h post-infiltration, GFP fluorescence was detected using an Olympus FV3000 confocal laser scanning microscope (excitation at 484 nm, emission at 507 nm).

## 5. Conclusions

This study presented a pan-genomic characterization of the *DXS* gene family across 14 Lauraceae genomes. We identified 92 proteins resolving into six orthogroups. We hypothesize that their evolutionary architecture might have been shaped by ancient WGD retention and recent SD or DD events. Clade 2 expanded to an average of 4.36 copies per genome, providing a genetic basis for diversified terpenoid metabolism. *DXS2*–*DXS4* represented retained ohnologs from the Lauraceae-specific WGD, whereas *DXS5* and *DXS6* contained recent genome-specific duplicates. Although all *DXS* genes were under purifying selection, subclades of Clade 2 exhibited distinct selection intensities. Sequence variation was largely confined to UTRs and introns, consistent with conservation of the catalytic core. Expression profiling in *C. officinarum* demonstrated tissue-specific partitioning: *CoDXS6* was highly expressed in developing leaves and fruits, *CoDXS2* was fruit-specific, and *CoDXS4* was flower-bud-specific. *CoDXS6* transcript abundance was significantly correlated with monoterpenoid accumulation (*r* = 0.945, *p* < 0.001). Functional validation confirmed the catalytic activity of *CoDXS6* and its positive modulation of monoterpenoid biosynthesis. *CoDXS6* was upregulated 6.32- to 9.18-fold in terpenoid-dominant chemotypes compared with phenylpropanoid-dominant chemotypes. Together, these findings uncovered evolutionary mechanisms underlying *DXS* diversification in Lauraceae and identified *CoDXS6* as a core regulator of leaf monoterpenoid biosynthesis. This gene may serve as a promising candidate target for future genetic improvement of essential-oil yield and composition via molecular breeding and metabolic-engineering strategies.

## Data Availability

The RNA-seq data of five organs (flower buds, bark, twigs, roots, and leaves), and leaves at four developmental stages have been deposited in the CNGB Sequence Archive (CNSA) of China National GeneBank DataBase (CNGBdb) under Bioproject accession numbers CNP0005973 and CNP0005950. The RNA- sequencing data for fruits at five developmental stages and the T2T genome of borneol-type *C. officinarum* are available from the corresponding author upon reasonable request.

## Supplementary Materials

The following supporting information can be downloaded at https://www.mdpi.com/article/10.3390/plants15182886/s1, Supplementary Table S1. The characteristics of 92 DXS proteins from 14 Lauraceae genomes. Supplementary Table S2. A total of 16 pairs of paralogous *DXS* genes were detected across 14 Lauraceae genomes originating from WGD or segmental duplications. Supplementary Table S3. Characterization of structural variations in *DXS* genes across 14 Lauraceae genomes, based on comprehensive whole-genome comparisons between the T2T genome of borneol-type *C. officinarum* and the other 13 Lauraceae genomes. Supplementary Table S4. TPM values of *DXS* genes derived from transcriptomes of different tissues and developmental stages of *C. officinarum*. Supplementary Table S5. Essential oils profile of fully expanded tender leaves from 7 chemotypes of *C. officinarum*. Supplementary Table S6. Primers for gene cloning, expression vector construction and qRT-PCR analysis. Supplementary Figure S1. Circos plot illustrating the chromosomal locations and intraspecific paralogous relationships of *DXS* loci across 13 additional Lauraceae genomes. Red lines denote paralogous gene pairs of *DXS2*–*DXS4*, while orange lines indicate other *DXS* paralogous pairs derived from whole-genome duplication (WGD) or segmental duplications. Grey lines represent other paralogous gene pairs within collinear blocks. Supplementary Figure S2. Total ion current chromatograms (TIC) from GC-MS analysis of FTL essential oils obtained from seven chemotypes of *C. officinarum*. Supplementary Figure S3. Multiple sequence alignment of the CoDXS family. Asterisks denote key conserved residues essential for DXS function. Numbers mark sites with amino acid substitutions in CoDXS3, including residues for catalytic activity (H142), GAP-substrate binding (H111, Y468), the Transket-pyr binding domain (G213, D214, A216, N243, Q245 and L248), and the Transketolase-C domain (D495, R496).

## Abbreviations

The following abbreviations are used in this manuscript:

ABA	abscisic acid
CDSs	coding sequences
CNV	copy number variation
CRE	cis-regulatory element
DD	dispersed duplication
DMAPP	dimethylallyl diphosphate
DPA	days post-anthesis
DXP	1-deoxy-D-xylulose 5-phosphate
DXS	1-deoxy-D-xylulose 5-phosphate synthase
eGFP	enhanced green fluorescent protein
FML	fully sclerified mature leaves
FTL	fully expanded tender leaves
GAP	glyceraldehyde 3-phosphate
GC-MS	gas chromatography-mass spectrometry
gDNA	genomic DNA
GRAVY	grand average of hydropathicity
HMM	hidden Markov model
HS-SPME	headspace solid-phase microextraction
IPP	isopentenyl diphosphate
IDI	isopentenyl-diphosphate delta-isomerase
*Ka*	non-synonymous substitution rate
kDa	kilodaltons
kb	kilobase
*Ks*	synonymous substitution rate
LB	Luria–Bertani
MEP	2-C-methyl-D-erythritol 4-phosphate pathway
ML	maximum likelihood
MVA	mevalonate pathway
Mya	million years ago
NYL	newly expanded young leaves
ORFs	open reading frames
PAV	presence/absence variation
pI	isoelectric point
qRT-PCR	quantitative real-time PCR
SD	segmental duplication
SV	structural variation
T2T	telomere-to-telomere
TD	tandem duplication
TPM	transcripts per kilobase of exon model per million mapped reads
TPP	thiamine diphosphate
TPS	terpene synthase
Transket-pyr	transketolase-pyrimidine
UTR	untranslated region
WGD	whole-genome duplication
