# Supplementary material for: Analysis of the DXS Pan-Gene Family Reveals Evolutionary Conservation and Regulatory Divergence Underpinning Terpenoid Metabolism in Lauraceae
Source: Plants (Basel). 2026 Sep 21;15(18):2886. doi: 10.3390/plants15182886 (PMC13610720; doi:10.3390/plants15182886)
Supplement: Supplementary file 1 [file plants-15-02886-s001.zip › plants-4497324-supplementary figures.pdf]

Article

Analysis of the DXS Pan-Gene Family Reveals Evolutionary Conservation and Regulatory Divergence Underpinning Terpenoid Metabolism in Lauraceae

Chao Fu [1,†], Siyang Tao [1,2,†], Shifang Wen [1] and Xindong Wang [1,*]

[1] Jiangxi Provincial Key Laboratory of Improved Variety Breeding and Efficient Utilization of Native Tree Species (NO. 2024SSY04091), Jiangxi Academy of Forestry, Nanchang 330032, China; plantfuchao@163.com (C.F.); taosy0822@163.com (S.T.); wenshifang@jxlky.cn (S.W.)

[2] College of Forestry, Jiangxi Agricultural University, Nanchang 330045, China

[*] Correspondence: x_wangxindong@jxlky.cn; Tel.: +86-0791-83987692

[†] These authors contributed equally to this work.

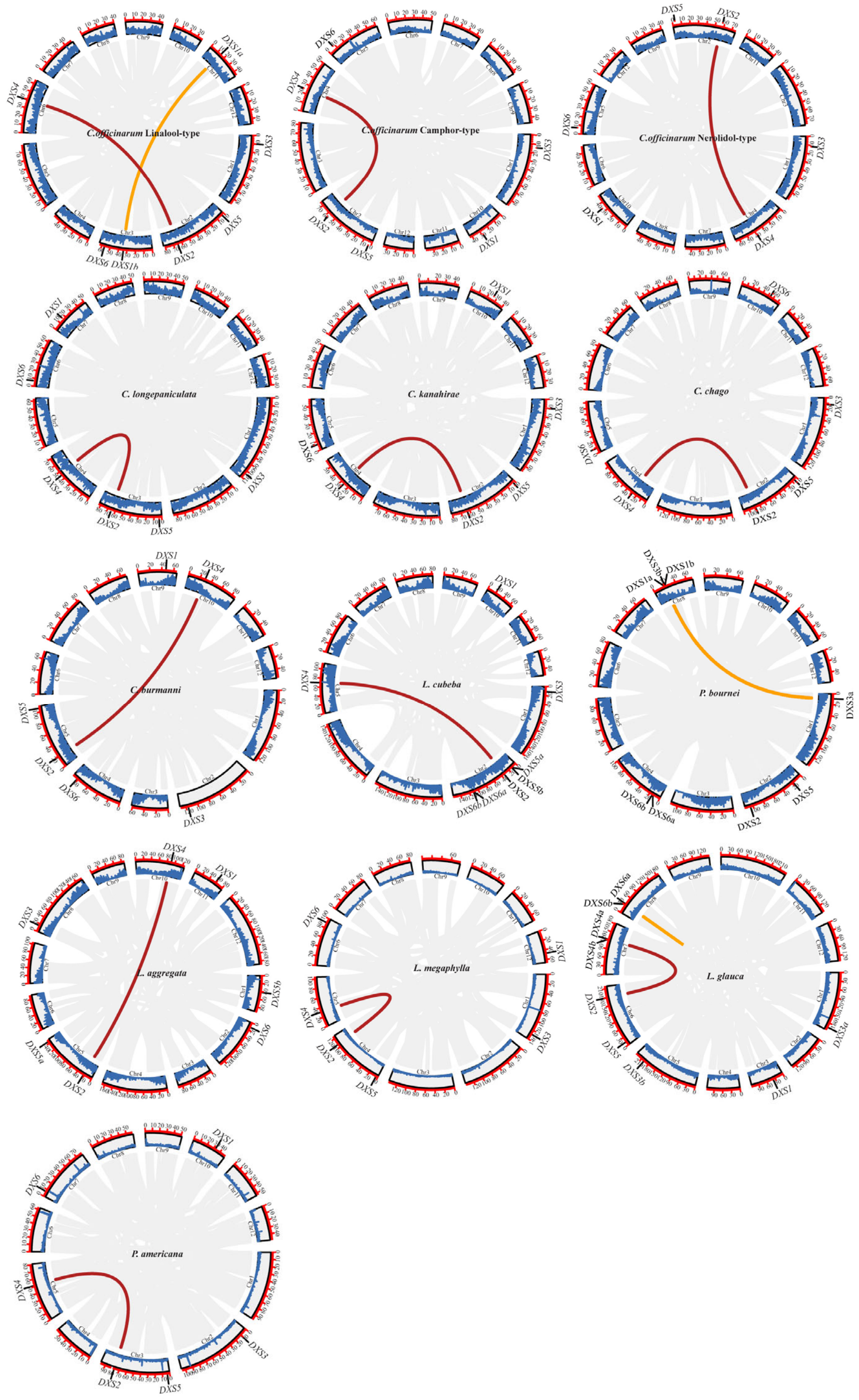

Supplementary Figure S1. Circos plot illustrating the chromosomal locations and intraspecific paralogous relationships of *DXS* loci across 13 additional Lauraceae genomes. Red lines denote paralogous gene pairs of *DXS2*–*DXS4*, while orange lines indicate other *DXS* paralogous pairs derived from whole-genome duplication (WGD) or segmental duplications. Grey lines represent other paralogous gene pairs within collinear blocks.

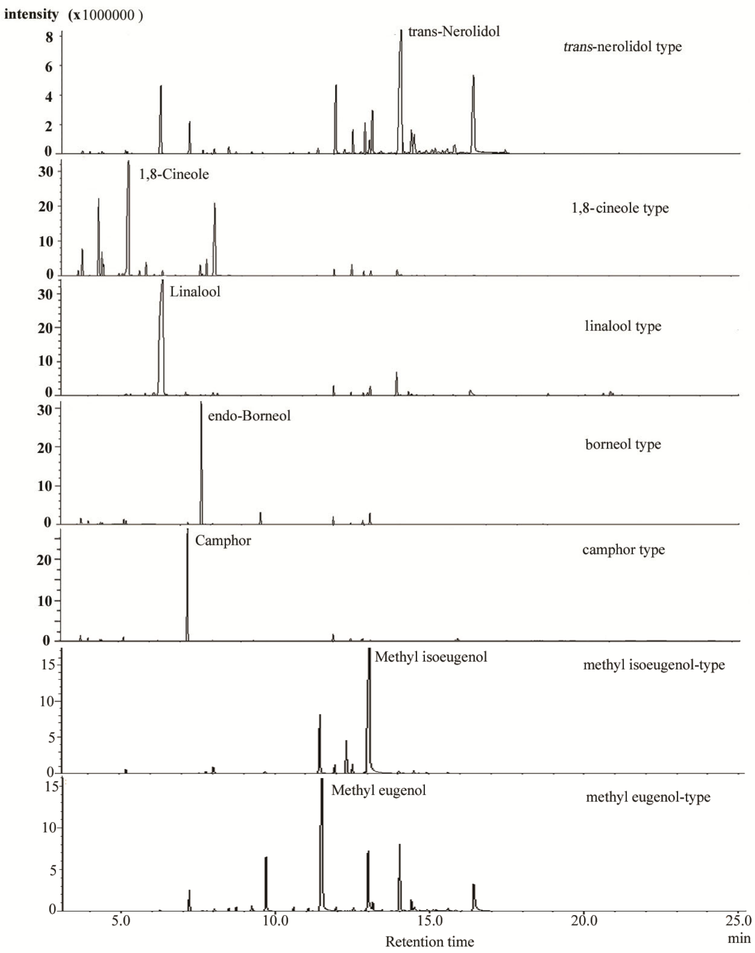

Supplementary Figure S2. Total ion current chromatograms (TIC) from GC-MS analysis of FTL essential oils obtained from seven chemotypes of *C. officinarum*.

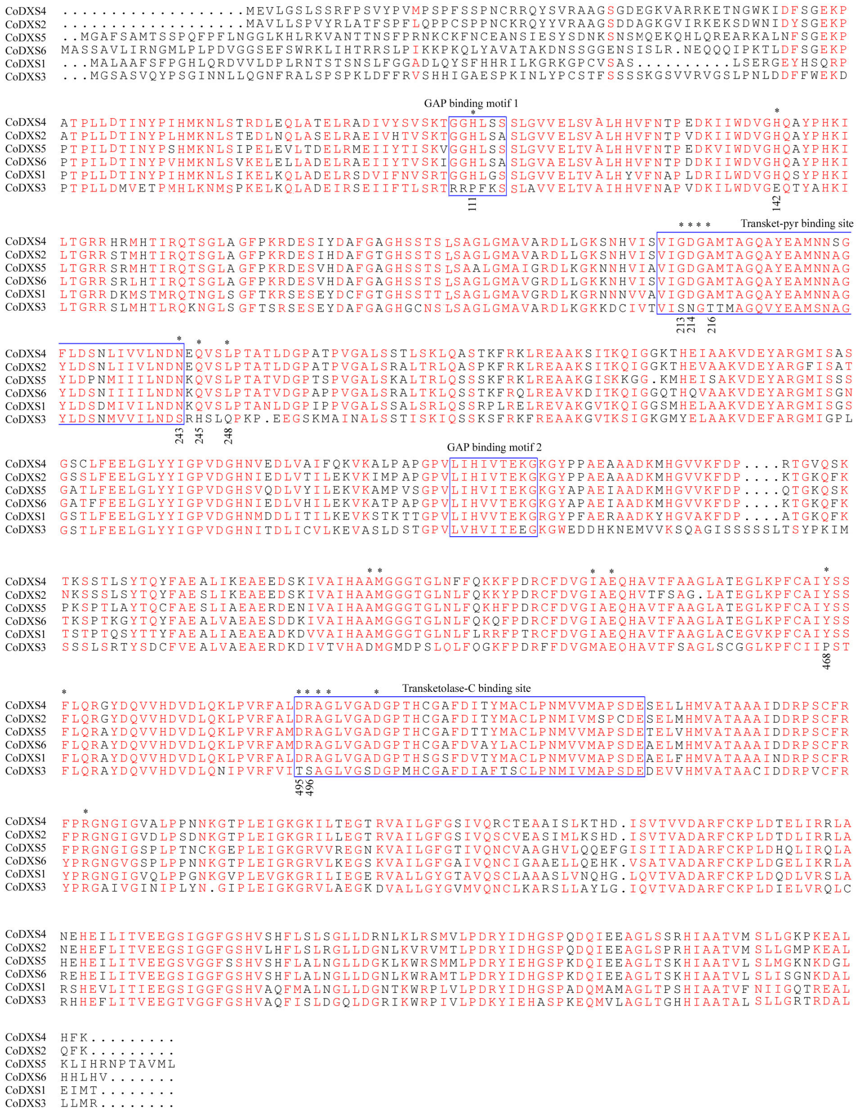

Supplementary Figure S3. Multiple sequence alignment of the CoDXS family. Asterisks denote key conserved residues essential for DXS function. Numbers mark sites with amino acid substitutions in CoDXS3, including residues for catalytic activity (H142), GAP-substrate binding (H111, Y468), the Transket-pyr binding domain (G213, D214, A216, N243, Q245 and L248), and the Transketolase-C domain (D495, R496).
